# Association of the neutrophil-to-platelet ratio with response to electroconvulsive therapy in adolescents with major depressive disorder

**DOI:** 10.3389/fpsyt.2024.1413608

**Published:** 2024-11-25

**Authors:** Dandan Geng, Wenxin Wang, Ning Du, Lisa Cynthia Niwenahisemo, Heyan Xu, Yuna Wang, Li Kuang

**Affiliations:** ^1^ Department of Psychiatry, The First Affiliated Hospital of Chongqing Medical University, Chongqing, China; ^2^ Mental Health Center, the First Affiliated Hospital of Chongqing Medical University, Chongqing, China; ^3^ The First Clinical College of Chongqing Medical University, Chongqing, China; ^4^ Mental Health Center, University-Town Hospital of Chongqing Medical University, Chongqing, China

**Keywords:** major depressive disorder, electroconvulsive therapy, adolescents, neutrophil-to-platelet ratio, response

## Abstract

**Background:**

Major depressive disorder (MDD) is one of the most serious mental disorders affecting adolescents worldwide. Electroconvulsive therapy (ECT) is widely acknowledged as a first-line treatment for severe depression, but the clinical response varies. Neutrophils and platelets are both related to the progression of MDD. The aim of this study was to investigate the correlation between the neutrophil-to-platelet ratio (NPR) during the acute phase and the effectiveness of ECT treatment.

**Methods:**

A total of 138 adolescent MDD patients who received ECT were included in the study. Neutrophil and platelet levels were obtained upon admission. At the same time, treatment response was the primary outcome measure, defined as a reduction of ≥ 50% in the HAMD-17 score from baseline to treatment endpoint, and the secondary outcome measure was remission of depression, defined as a HAMD-17 score ≤ 7.

**Results:**

After receiving ECT, 103(74.6%) of all patients responded to treatment and 72(52.2%) achieved remission. Non-responders/non-remitters to ECT tended to have higher levels of NPR at baseline compared to ECT responders/remitters [Non-responder: 3.4 (2.5-4.8) vs 2.7 (2.2-3.5), P = 0.002; Non-remitter: 0.014 (0.011-0.017) vs 0.011 (0.008-0.015), P = 0.03]. In multiple logistic regression, high NPR (≥ 0.014) remained independently associated with ECT non-response/non-remission after adjusting for confounding factors [Non-responder: OR = 4.911, 95% CI (2.052 - 11.754), P < 0.001; Non-remitter: OR = 2.704, 95% CI (1.262 - 5.796), P = 0.011].

**Conclusion:**

High NPR correlates with poor ECT efficacy in adolescents with MDD, particularly among female and overweight patients.

## Introduction

Adolescence constitutes a pivotal phase in the advancement and preservation of emotional intelligence, playing a vital role in mental health. However, this period is also vulnerable to mood disorders, such as major depressive disorder (MDD), which is recognized as the most severe psychological condition impacting adolescents globally (1, 2). The defining feature of MDD is the presence of intense and recurring episodes of depression. In contrast to adults, adolescents with mood disorders demonstrate more pronounced functional impairment and diminished quality of life, along with a protracted disease duration, reduced likelihood of remission, and heightened susceptibility to relapse (3). The lifetime prevalence of MDD is 11.1-14.6%, with a cumulative prevalence of up to 20% by the end of adolescence (4, 5). During adolescence, there is an increase in suicidal ideation and self-harm behavior, and 61% of teens who attempt suicide have mood disorders (1). It is crucial to understand the neuropathological basis of MDD adolescents and to identify for risk factors and predictors that influence its prognosis.

Over the last 80 years, electroconvulsive therapy (ECT) has consistently been one of the most effective approach for addressing acute major depressive episodes. Even for patients with treatment-resistant depression, ECT achieves remission rates of up to 75% (6, 7). Given the high cost of ECT, the potential side effects associated with it, and the fact that approximately one-third of patients do not respond to ECT treatment (8), it is crucial to differentiate patients with depression who are most likely to benefit from ECT, which is critical for appropriate treatment and better outcomes for patients. Unfortunately, clinical variables are of limited value in predicting ECT response.

In fact, a substantial body of research has extensively discussed the role of inflammation in depression (9–11). Evidence suggests that inflammation may contribute to depression by altering neurotransmission, hippocampal neurogenesis, neuroendocrine function, and reactive oxygen species (12, 13). These changes can potentially lead to cognitive impairment and the emergence of depressive symptoms. Additionally, research has demonstrated that in the majority of patients with MDD, inflammation is linked to a poor response to treatment with antidepressants (14). A meta-analysis revealed that individuals who responded to antidepressants had lower baseline levels of IL-8 and exhibited a greater reduction in TNF-α compared to non-responders (15). Another study on depressed patients found that higher baseline levels of IL-6 and CRP were correlated with more severe depressive symptoms five years later (16). Most of the current studies on MDD inflammation focus on cytokines or CRP (17), but these markers are not easy to obtain at the time of admission and require a certain economic cost.

Neutrophils are commonly utilized as a signal of systemic inflammation due to their affordability and easy accessibility. Transport of immune cells to sites of inflammation is regulated by neutrophils, which are the first immune cells to respond to inflammation (18). Numerous studies have demonstrated a link between neutrophils and neuropsychiatric disorders, such as bipolar disorder (19), depression (20), schizophrenia (21), autism spectrum disorder (22), and others. The involvement of neutrophils is crucial in the pathogenesis of depression (23). Their activation can lead oxidative stress by the release of ROS, which is also considered to be one of the pathophysiological mechanisms of depression (24).

Platelets are an indispensable part of thrombosis and hemostasis, as they are an important component of blood cells and maintain the integrity of the vascular endothelium (25). It is best-known that platelets also play an essential role in inflammation (26), they can stimulate the recruitment and extravasation of neutrophils into the brain parenchyma following acute stroke, which is associated with the progression of neuronal damage (27, 28). Numerous epidemiological and genetic evidence over the past few decades have demonstrated a connection between platelet levels in the brain and neurodegenerative/neuropsychiatric diseases such as Alzheimer’s disease (AD), Parkinson’s disease (PD), and MDD (29).

High neutrophil-platelet complexes may have something to do with the development of symptomatic carotid stenosis in stroke patients (30). Previous studies have shown the value of monitoring disease activity in ulcerative colitis using a neutrophil-to-platelet ratio (NPR), with good correlation, sensitivity, and specificity (31). A recent study found that the high NPR was linked to poor long-term outcomes in acute ischemic stroke (32). NPR has been suggested as an efficient and expedient screening instrument for evaluating systemic inflammation in infectious endocarditis (33). However, to date, no studies have been conducted to determine whether the baseline NPR is predictive of ECT treatment response in adolescents with MDD.

## Methods

### Subject

From May 2023 to November 2023, we recruited MDD patients admitted for treatment at the First Affiliated Hospital of Chongqing Medical University. Patients meeting the following criteria were included and completed this nested cohort study: (1) patients aged 13-18 years; (2) diagnosed with MDD according to DSM-V criteria; (3) Hamilton Depression Scale (HAMD-17) ≥ 17; (4) neutrophil and platelet levels were tested within 24 hours of admission; (5) received ECT treatment. Exclusion criteria were as follows: (1) history of manic or hypomanic episodes; (2) ECT in the previous 6 months; (3) previous history of brain disorders or severe traumatic brain injury; (4) patients with severe physical illnesses; (5) patients with active infections, autoimmune diseases, anti-inflammatory or immunosuppressive therapy; (6) history of substance dependence or abuse; (7) patients unable to complete the final follow-up. The research was approved by the Ethics Committee of the First Affiliated Hospital of Chongqing Medical University, per the ethical guidelines of the 1975 Helsinki Declaration (approval no. K2023-676). Each participant or their guardian signed an informed consent form after understanding the details of our study.

### Data collection

Clinical variables and demographic data were collected by trained psychiatrists, including age, gender, body mass index (BMI), current smoking and drinking habits, number of ECT sessions, concomitant medications, etc. Overweight was defined as BMI ≥ 25kg/m^2^. Additionally, the types of drugs patients received in the hospital were divided into: antidepressants [selective serotonin reuptake inhibitors (SSRIs), serotonin and norepinephrine reuptake inhibitors (SNRIs), serotonin antagonist and reuptake inhibitors (SARIs), norepinephrine-dopamine reuptake inhibitors (NaSSA)], antipsychotics (risperidone, olanzapine, quetiapine, or aripiprazole), and anxiolytics (benzodiazepines and z-drug hypnotics). Fasting blood samples were collected from each participant within 24 hours of admission and measured for neutrophil and platelet counts using the Sysmex XN 1500 hematology analyzer. NPR was figured up as the ratio of neutrophil count to platelet count.

### Clinical assessment

HAMD-17 was finished and collected within 24 hours before the first ECT treatment and within 1 week after ECT completion. Treatment response was defined as the primary outcome measure, with a ≥ 50% reduction in HAMD-17 scores from baseline to post-ECT. Remission of depression, defined as HAMD-17 scores ≤ 7, was considered a secondary outcome measure (34). The percentage change in HAMD-17 scores from baseline to the end of ECT was used as a continuous outcome measure.

### Electroconvulsive therapy procedure

After fully understanding the adverse reactions and benefits of ECT, all patients and their legal guardians voluntarily consent to the treatment, with informed consent signed by the legal guardians. Before the treatment, all doctors in the patient’s medical team will discuss and evaluate the indications and contraindications of ECT. During the treatment, patients have the right to request to stop ECT at any time. All ECT treatments are conducted using the Thymatron DGx system (Somatics LLC, Lake Bluff, Illinois, USA) with bilateral temporal electrode placement in brief pulse mode. The initial electrical dose is determined by the formula of age multiplied by 0.7, and subsequent stimulus energy is adjusted based on seizure duration. If the seizure duration is less than 25 seconds, the energy is increased by 5% in the next treatment (35). Propofol (1.5-2 mg/kg) and succinylcholine (0.5-1 mg/kg) are used for anesthesia and muscle relaxation, with atropine administered as needed to regulate heart rate.

### Statistical analysis

Chi-square test or Fisher’s exact test was used to compare intergroup differences in categorical variables. T-test or Mann-Whitney U test was employed to assess differences in continuous variables. Additionally, receiver operating characteristic (ROC) curves were used to evaluate NPR’s specificity, sensitivity, and cut-off values. The area under the ROC curve (AUC) was used to test the predictive value of the indicator. Multivariate logistic regression models were applied to assess the association between baseline NPR and ECT response, adjusted for confounding factors, including age, gender, BMI, number of ECT sessions, antidepressants, antipsychotics, anxiolytics, that have been associated with depression prognosis in previous studies. Finally, linear regression analysis was conducted to examine whether the influence of NPR on the efficacy of ECT in adolescent MDD patients differed across subgroups. A P-value < 0.05 was considered statistically significant. All data analyses were performed using IBM SPSS Statistics software Version 26 for Windows.

## Results

### General information

From May 2023 through November 2023, a total of 209 adolescent patients with MDD were recruited from the First Affiliated Hospital of Chongqing Medical University. 71 patients were excluded for not meeting the criteria, including 34 patients with manic or hypomanic episodes, 12 patients who received ECT in the previous 6 months, 10 patients who did not receive ECT treatment, and 15 patients who could not complete the final scale assessment. Ultimately, 138 adolescent patients with MDD were included and completed this study. Among the 138 cases of adolescent MDD patients, there were 98 females (68.1%) and 44 males (31.9%), with an average age of 14.5 ± 1.3 and an average BMI of 21.5 ± 3.6. After receiving ECT, there were 103 responders (74.6%) and 72 remitters (52.2%) in all patients (**Table 1**).

**Table 1:** Baseline characteristics of the samples under study.

Variables	All patients (n=138)						
	Total	Responders (n = 103)	Non-Responders (n = 35)	P-value	Remitters (n =72)	Non-Remitters (n = 66)	P-value
Age, year (mean ± SD)	14.5 ± 1.3	14.5 ± 1.3	14.7 ± 1.2	0.437	14.5 ± 1.3	14.5 ± 1.2	0.838
Female, n (%)	94 (68.1%)	69 (67.0%)	25 (71.4%)	0.626	43 (60.6%)	51 (76.1%)	0.050
BMI, kg/m^2^ (mean ± SD)	21.5 ± 3.6	21.5± 3.6	21.3 ± 3.5	0.765	21.5 ± 3.5	21.5 ± 3.6	0.956
Overweight, n (%)	31 (22.5%)	22 (21.4%)	9 (25.7%)	0.594	16 (22.5%)	15 (22.4%)	0.983
Currently smoking, n (%)	15 (10.9%)	12 (11.7%)	3 (8.6%)	0.613	9 (12.7%)	6 (9.0%)	0.483
Currently drinking, n (%)	14 (10.1%)	11 (10.7%)	3 (8.6%)	0.721	7 (9.9%)	7 (10.4%)	0.909
Medications, n (%)							
SSRI, n (%)	95 (68.8%)	69 (67.0%)	26 (74.3%)	0.421	49 (69.0%)	46 (68.7%)	0.964
SNRI, n (%)	30 (21.7%)	20 (19.4%)	10 (28.6%)	0.257	13 (18.3%)	17 (25.4%)	0.315
Mirtazapin,n (%)	12 (8.7%)	8 (7.8%)	4 (11.4%)	0.507	4 (5.6%)	8 (11.9%)	0.189
Trazodone,n (%)	4 (2.9%)	3 (2.9%)	1 (2.9%)	0.987	1 (1.4%)	3 (4.5%)	0.283
Antipsychotics, n (%)	114 (82.6%)	84 (81.6%)	30 (85.7%)	0.575	61 (85.9%)	53 (79.1%)	0.291
Benzodiazepine, n (%)	70 (50.7%)	50 (48.5%)	20 (57.1%)	0.379	38 (53.5%)	32 (47.8%)	0.499
Z-drug hypnotics, n (%)	51 (37.0%)	33 (32.0%)	18 (51.4%)	0.040*	23 (32.4%)	28 (41.8%)	0.253
Number of ECT sessions, (median, IQR)	7.0 (6.0-8.0)	7.0 (6.0-8.0)	7.0 (6.0-8.0)	0.922	7.0 (6.0-8.0)	7.0 (6.0-8.0)	0.208
Baseline HAMD-17, (median, IQR)	33.0 (28.0-38.3)	33.0 (27.0-38.0)	33.0 (29.0-40.0)	0.366	31.0 (27.0-38.0)	35.0 (29.0-40.0)	0.112
Post-ECT HAMD-17, (median, IQR)	7.0 (6.0-15.0)	7.0 (6.0-11.0)	21.0 (7.0-24.0)	< 0.001*	6.0 (5.0-7.0)	15. 0(11.0-22.0)	< 0.001*
Platelets (10*9/L), mean (SD)	236.8 ± 59.6	243.2 ± 61.8	218.1 ± 49.1	0.017*	242.7 ± 59.0	230.6 ± 60.1	0.236
Neutrophils (10*9/L), (median, IQR)	2.7 (2.2-3.8)	2.7 (2.2-3.5)	3.4 (2.5-4.8)	0.002*	2.6 (2.2-3.3)	3.2 (2.2-4.3)	0.027*
NPR, median (IQR)	0.012 (0.009-0.016)	0.011 (0.009-0.015)	0.016 (0.011-0.021)	< 0.001*	0.011 (0.008-0.015)	0.014 (0.011-0.017)	0.003*

BMI, body mass index; SSRI, selective serotonin reuptake inhibitor; SNRI, serotonin-norepinephrine reuptake inhibitor; ECT, electroconvulsive therapy; HAMD-17, Hamilton depression rating scale, 17-item version; NPR, neutrophil-to-platelet ratio.

*P values considered statistically significant; *P < 0.05.

### Demographic and clinical characteristics

We assessed the baseline characteristics of ECT responders and non-responders, as well as ECT remitters and non-remitters (**Table 1**). In any ECT subgroup, there were no differences in age, gender, BMI, overweight, current smoking and drinking, number of ECT sessions, and baseline HAMD-17 score. Compared with ECT responders, fewer adolescent patients in the ECT non-responders received z-drug hypnotics (51.4% vs 32.0%, P = 0,040). There were no significant differences in the use of other drugs such as antidepressants and antipsychotics among any ECT subgroup. The baseline neutrophil count (median (IQR): 3.4 (2.5-4.8) vs 2.7 (2.2-3.5), P = 0.002) and NPR (median (IQR): 0.016 (0.011-0.021) vs 0.011 (0.009-0.015), P < 0.001) were markedly higher in the ECT non-responders than in the ECT responders. However, the baseline platelet count in the ECT non-responders group was substantially lower than that in the ECT responders (mean ± SD: 218.1 ± 49.1 vs 243.2 ± 61.8, P = 0.017). Similar findings were also observed between ECT remitters and non-remitters. Compared with ECT remitters, ECT non-remitters tended to have higher levels of neutrophils at baseline (median (IQR): 3.2 (2.2-4.3) vs 2.6 (2.2-3.3), P = 0.027) and higher levels of NPR (median (IQR): 0.014 (0.011-0.017) vs 0.011 (0.008-0.015), P = 0.03). However, there was no significant difference in baseline platelet levels between the ECT remitters and non-remitters. In addition, the proportion of females in the ECT non-remitters group was higher than that in the ECT remitters group, although not reaching statistical significance (76.1% vs 60.6%, P = 0.05).

### Baseline characteristics of different NPR groups in adolescent MDD patients

**Figure 1:**
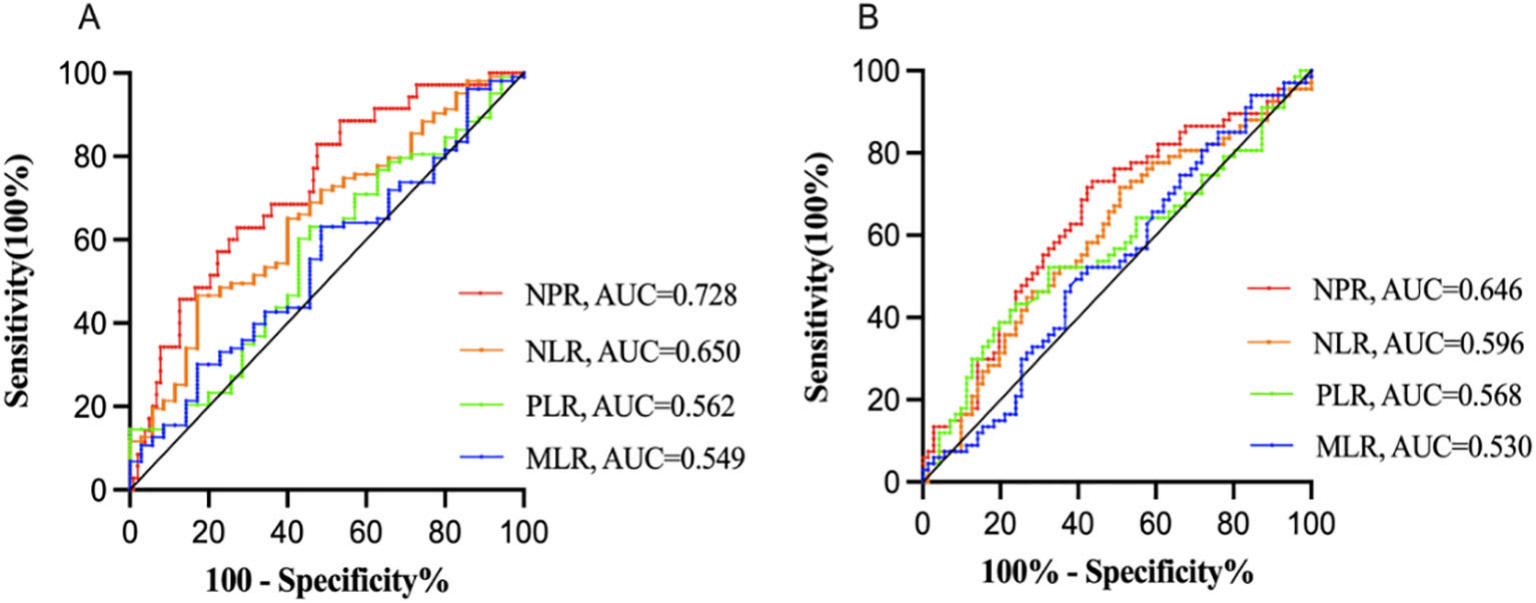
Receiver operator characteristic (ROC) analysis of blood test ratios for prediction of ECT response **(A)** and remission **(B)** NPR, neutrophil-to-platelet ratio; NLR, neutrophil-to-lymphocyte ratio; PLR, platelet-to-lymphocyte ratio; MLR, monocyte-to-lymphocyte ratio; AUC, area under the curve. Compared with other blood cell ratios such as NLR, PLR and MLR, NPR exhibited greater discriminative ability in predicting ECT response/remission in adolescent MDD patients, with AUC of 0.728 (P < 0.001) **(A)** and 0.646 (P = 0.003) **(B)**, respectively.

According to the ROC curve analysis, compared with other blood cell ratios such as neutrophil-to-lymphocyte ratio (NLR) [Response: AUC = 0.650, 95% CI (0.547-0.752), P = 0.008; Remission: AUC = 0.596, 95% CI (0.500 - 0.691), P = 0.051], platelet-to-lymphocyte ratio (PLR) [Response: AUC = 0.562, 95% CI (0.452-0.672), P = 0.270; Remission: AUC = 0.568, 95% CI (0.471-0.665), P = 0.163], and monocyte-to-lymphocyte ratio (MLR) [Response: AUC = 0.549, 95% CI (0.440-0.657), P = 0.270; Remission: AUC = 0.530, 95% CI (0.433-0.627), P = 0.535], NPR exhibited greater discriminative ability in predicting ECT response/remission in adolescent MDD patients, with AUC of 0.728 (P < 0.001) (**Figure 1A**) and 0.646 (P = 0.003) (**Figure 1B**), respectively. Furthermore, NPR predicted ECT response with a specificity of 62.9%, sensitivity of 72.8%, and optimal cut-off point of 0.014.

Based on the optimal cut-off value, NPR was divided into two groups, low NPR (< 0.014) and high NPR (≥ 0.014). As shown in **Table 2**, compared with the low NPR group, the high NPR group had higher BMI (mean ± SD: 22.6 ± 3.3 vs 21.3 ± 3.6, P = 0.039), more overweight patients (40.0% vs 12.5%, P < 0.001), fewer ECT responders (56.0% vs 85.2%, P < 0.001) and remitters (36.0% vs 60.2%, P = 0.006).

**Table 2:** Baseline characteristics of patients with depression in different NPR groups.

Variables	NPR		P-value
	Low-NPR (n = 88) (< 0.014)	High-NPR (n = 50) (≥ 0.014)	
Age, year (mean ± SD)	14.5 ± 1.3	14.4 ± 1.3	0.812
Female, n (%)	58 (65.9%)	36 (72.0%)	0.461
BMI, kg/m^2^(mean ± SD)	21.3 ± 3.6	22.6 ± 3.3	0.039*
Overweight, n (%)	11 (12.5%)	20 (40.0%)	< 0.001*
Currently smoking, n (%)	8 (9.1%)	7 (14.0%)	0.373
Currently drinking, n (%)	10 (11.4%)	4 (8.0%)	0.529
Medications, n (%)			
SSRI, n (%)	60 (68.2%)	35 (70.0%)	0.825
SNRI, n (%)	16 (18.2%)	14 (28.0%)	0.179
Mirtazapin,n (%)	10 (11.4%)	2 (4.0%)	0.140
Trazodone,n (%)	2 (2.3%)	2 (4.0%)	0.561
Antipsychotics, n (%)	72 (81.8%)	42 (84.0%)	0.745
Benzodiazepine, n (%)	46 (52.3%)	24 (48.0%)	0.629
Z-drug hypnotics, n (%)	28 (31.8%)	23 (46.0%)	0.097
Number of ECT sessions, (median, IQR)	7.0 (6.0-8.0)	7.0 (6.0-8.0)	0.070
Baseline HAMD-17, (median, IQR)	33.0 (29.0-38.0)	33.0 (27.0-39.3)	0.787
Post-ECT HAMD-17, (median, IQR)	10.5 (7.0-15.0)	15.5 (10.8-21.0)	< 0.001*
Responders, n (%)	75 (85.2%)	28 (56.0%)	< 0.001*
Remitters, n (%)	53 (60.2%)	18 (36.0%)	0.006*

NPR, neutrophil-to-platelet ratio; BMI, body mass index; SSRI, selective serotonin reuptake inhibitor; SNRI, serotonin-norepinephrine reuptake inhibitor; ECT, electroconvulsive therapy; HAMD-17, Hamilton depression rating scale, 17-item version.

*P values considered statistically significant; *P < 0.05.

### Association between baseline NPR and ECT response/remission

In multivariable logistic regression analyses, we used ECT response and remission as dependent variables and low NPR (< 0.014) as the reference for two regression models. After adjustment for potential confounders including age, sex, BMI, number of ECT sessions, antidepressants, antipsychotics, and anxiolytics, high NPR remained independently associated with ECT non-response/non-remission [Non-response: OR = 4.911, 95% CI (2.052-11.754), P < 0.001 (**Table 3**); Non-remission: OR = 2.704, 95% CI (1.262-5.796), P = 0.011 (**Table 4**)].

**Table 3:** Multivariate logistic regression analysis for the association between NPR and ECT response.

	Model 1		Model 2	
	OR (95% CI)	*P*-value	OR (95% CI)	*P*-value
NPR				
Low-NPR	**Reference**		**Reference**	
High-NPR	5.045 (2.163-11.766)	< 0.001*	4.911 (2.052-11.754)	< 0.001*
Age	1.145 (0.803-1.580)	0.408	1.150 (0.821-1.611)	0.417
Female	1.210 (0.489-2.998)	0.680	1.294 (0.514-3.260)	0.585
BMI	0.936 (0.828-1.059)	0.294	0.960 (0.841-1.096)	0.546
Number of ECT sessions			0.932 (0.749-1.158)	0.523
Antidepressants			2.730 (0.518-14.389)	0.236
Antipsychotics			0.791 (0.270-2.318)	0.453
Anxiolytics			1.291 (0.525-3.173)	0.578

NPR, neutrophil-to-platelet ratio; BMI, body mass index; ECT, electroconvulsive therapy; OR, odds ratio; CI, confidence interval.

Model 1: adjusted for age, gender, and BMI.

Model 2: adjusted for variables in Model 1 plus number of ECT sessions, antidepressants, antipsychotic, and anxiolytics.

*P values considered statistically significant; *P < 0.05.

**Table 4:** Multivariate logistic regression analysis for the association between NPR and ECT remission.

	Model 1		Model 2	
	OR (95% CI)	P-value	OR (95% CI)	P-value
NPR				
Low-NPR	**Reference**		**Reference**	
High-NPR	2.772 (1.318-5.829)	0.007*	2.704 (1.262-5.796)	0.011*
Age	1.041 (0.789-1.374)	0.756	1.040 (0.783-1.382)	0.785
Female	2.082 (0.970-4.467)	0.060	2.031 (0.941-4.384)	0.071
BMI	0.968 (0.874-1.072)	0.529	0.982 (0.875-1.103)	0.762
Number of ECT sessions			1.051 (0.872-1.267)	0.601
Antidepressants			1.429 (0.442-4.624)	0.551
Antipsychotics			1.436 (0.558-3.691)	0.453
Anxiolytics			0.833 (0.394-1.761)	0.632

NPR, neutrophil-to-platelet ratio; BMI, body mass index; ECT, electroconvulsive therapy; OR, odds ratio; CI, confidence interval.

Model 1: adjusted for age, gender, and BMI.

Model 2: adjusted for variables in Model 1 plus number of ECT sessions, antidepressants, antipsychotic, and anxiolytics.

*P values considered statistically significant; *P < 0.05.

### Differences in the correlation between NPR and ECT efficacy

**Figure 2:**
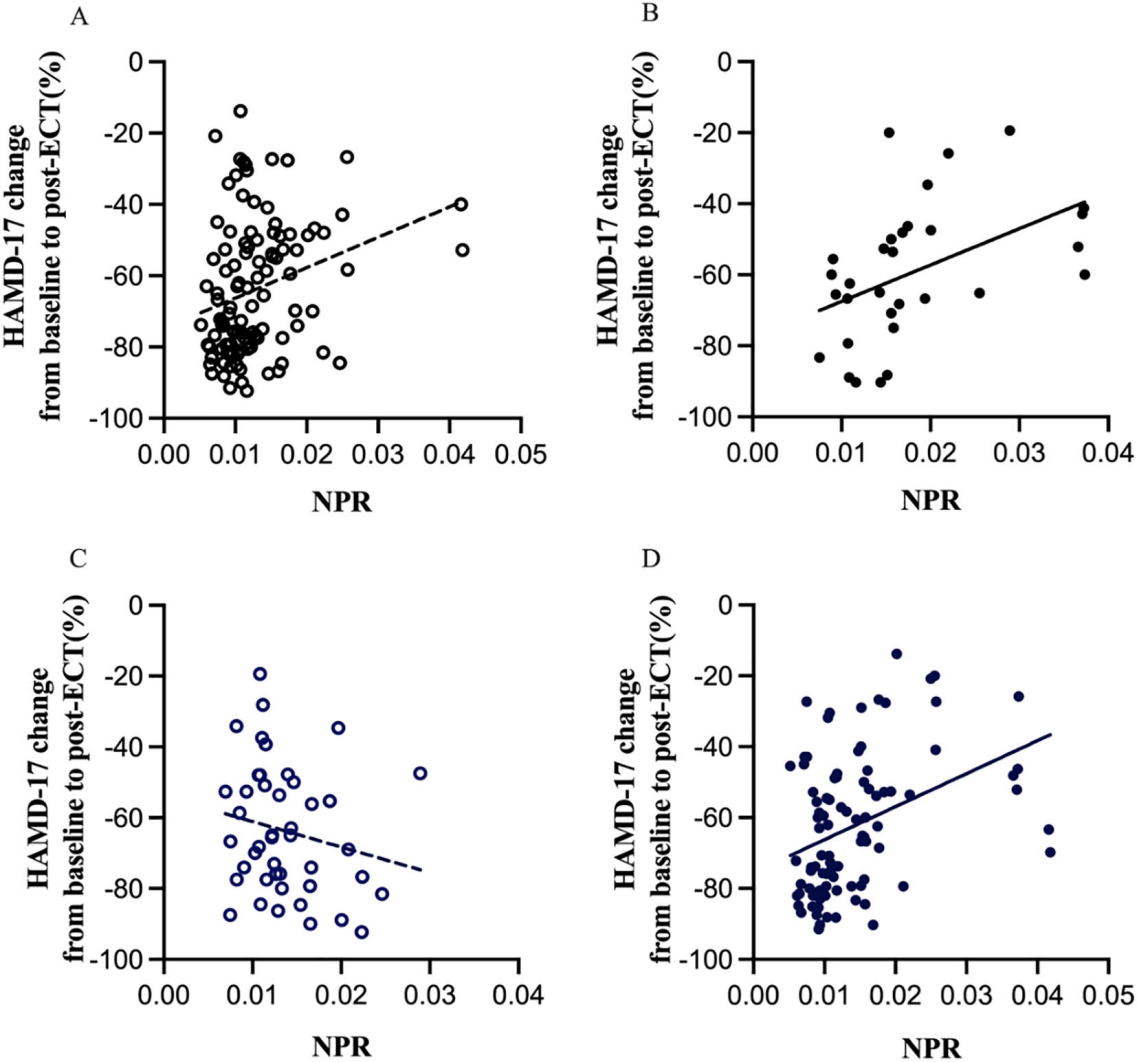
Subgroup analysis indicating the correlations between NPR and the percentage change in HAMD-17 scores from baseline to post-ECT. NPR, neutrophil-to-platelet ratio; ECT, electroconvulsive therapy; HAMD-17, Hamilton depression rating scale, 17-item version. Linear regression analyses showed that baseline NPR was positively correlated with the percentage difference in HAMD-17 scores in non-overweight MDD patients (r = 0.274, p = 0.004) **(A)**, whereas in overweight MDD patients, the correlation was stronger (r = 0.462, p = 0.009) **(B)**. Moreover, in female patients, NPR was positively correlated with the percentage change in HAMD-17 scores (r = 0.380, P < 0.001) **(D)**, whereas there was no correlation observed in male patients (r = -0.194, P = 0.208) **(C)**.

Linear regression analyses showed that the relationship between baseline NPR level and the percentage change in HAMD-17 score from baseline to post-ECT varied in adolescent MDD patients with or without overweight and by sex. As shown in **Figure 2A**, baseline NPR was positively correlated with the percentage difference in HAMD-17 scores in non-overweight MDD patients (r = 0.274, P = 0.004). In contrast, in overweight MDD patients, the correlation was stronger (r = 0.462, P = 0.009) (**Figure 2B**). Moreover, in female patients, baseline NPR was positively correlated with the percentage change in HAMD-17 scores (r = 0.380, P < 0.001) (**Figure 2D**), whereas there was no correlation observed in male patients (r = -0.194, P = 0.208) (**Figure 2C**).

## Discussion

The initial goal of this study was to explore a new accurate biomarker that could predict the efficacy of ECT in adolescents with MDD. Due to the high cost and potential side effects of ECT, it is of great clinical significance to identify an easily accessible and inexpensive biomarker during the acute phase to determine adolescents with MDD who are most likely to benefit from ECT. This study demonstrated that high baseline NPR is associated with a lower response rate and remission rate after ECT in adolescents with MDD. Our findings revealed that NPR has better predictive value compared to other blood markers such as NLR, PLR, and MLR. Furthermore, we also found that the predictive effect of NPR may depend on different conditions, especially in female and overweight adolescent patients.

Neutrophils, the largest component of white blood cells, are related to the occurrence and development of depression as they are the immune cells that respond earliest to inflammation (36, 37). In a meta-analysis that included 104 studies, it was found that people with depression had significantly higher neutrophil counts in their blood and cerebrospinal fluid than healthy individuals (38). Another study showed that neutrophil counts were also significantly elevated in depressed patients who had attempted suicide, a common feature of depression (39). Our study showed that non-responders and non-remitters to ECT often have higher levels of baseline neutrophils, consistent with previous research. One possible reason is that higher levels of neutrophils may reflect the severity of initial inflammation during the acute phase of MDD (34). Neutrophils can secrete inflammatory mediators, such as IL-6 and TNF-α, which can damage the brain’s neurotransmitter system and worsen the symptoms of depression (40). In addition, they can also release ROS, leading to oxidative stress response causing damage to brain cells, neuronal apoptosis, and consequently affect the outcome of depression (24).

Platelets have the function of maintaining vascular endothelial integrity (25). It is well known that platelets also play an important role in inflammation (26). Musselman et al. observed that patients affected by MDD showed increased baseline platelet activation and reactivity (41). Afsane Bahrami et al. reported that higher depression scores were associated with increased platelets (42). Another study by Moshui Shan et al. suggested that there might be a U-shaped relationship between platelets and the severity of depressive symptoms (43). However, unlike previous findings, our study showed a tendency of higher baseline platelet count in ECT responders, with no similar difference observed in ECT remitters. Possible explanations are that platelets not only reflect neurons in multiple aspects but also directly affect their pathophysiological changes in multiple ways (44). Brain-derived neurotrophic factor (BDNF), which regulates neural circuit development and function, is stored in platelets and released by activated platelets during aggregation at the injury site (45, 46). Higher platelet counts indicate higher concentrations of BDNF in the serum (47, 48), and the increase in BDNF helps restore neural function and regulate mood (49). Similarly, serotonin (5-HT) (a neurotransmitter that plays a crucial role in controlling behavior and social activity) is also stored in platelet-dense granules and can serve as a weak agonist upon release after activation (50, 51). Higher platelet counts may indicate relatively more active 5-HT function, and Marlene S Williams et al. reported that increased platelet 5-HT uptake is associated with a reduction in the severity of depression (52). Platelets are widely regarded as a peripheral model of neuronal activity in neuropsychology, and depletion of platelets may affect BDNF and serotonin level expression, thereby affecting the occurrence and development of depression (53–55).

This study explored the synergistic effect of neutrophils and platelets on adolescent MDD patients’ response to ECT by exploring NPR. According to our study, baseline high NPR was independently associated with the poor efficacy of ECT in adolescent MDD patients. The exact mechanisms involved cannot be determined at present. We speculate that on the one hand, the high baseline NPR may reflect the high inflammatory level of acute-phase patients, emphasizing the complex relationship between inflammation and disease progression. Increased inflammation may affect neurotransmitter transmission and even lead to neuronal apoptosis, thereby reducing the patients’ response capacity to ECT. On the other hand, this index also partially reflects the pathophysiological changes of neurons, suggesting a deficiency of BDNF and serotonin neurotransmitters that regulate neural circuits and development.

In addition, an interesting finding is that the predictive efficacy of NPR varies among different subgroups. We speculate that this difference may be due to the varying sensitivity to inflammation among different subgroups. Firstly, our study found that NPR is a stronger predictor of clinical outcomes in adolescent MDD patients receiving ECT, particularly in overweight adolescents. One possible explanation is that overweight/obese individuals experience a state of low-grade inflammation, known as “chronic low-grade inflammation”, which releases inflammatory factors and enhances the body’s inflammatory response (56, 57). Additionally, adipose tissue in overweight/obese individuals contains immune cells that also release inflammatory mediators, leading to an increased inflammatory response (58). Therefore, the inflammatory response in overweight/obese individuals is more pronounced. Secondly, baseline NPR in female adolescent patients is positively correlated with changes in HAMD-17 scores, while no correlation is observed in male patients. The possible reason is that during the inflammatory process, immune cell reactions in females are faster and stronger, leading to a more intense inflammatory response (58, 59). Furthermore, female susceptibility to inflammation is also influenced by hormones, physiological cycles, and genetic factors, which may enhance the inflammatory response (60). Although the molecular mechanisms supporting this theory are still to be explored, it is important to pay attention to special populations with a higher risk of poor response to ECT in adolescent MDD patients, such as females and overweight individuals.

Some limitations of this study need to be acknowledged. Firstly, our sample size is relatively small, limiting the generalizability of the findings to larger populations. The next step of our work should be to expand the sample size to confirm the relationship between NPR and ECT efficacy. Secondly, NPR was only recorded once at admission, and the dynamic changes of NPR during hospitalization and its relationship with ECT efficacy could not be obtained. In addition, we lack other relevant inflammatory markers such as CRP or interleukins to confirm the predictive role of NPR, which may partially reflect changes in inflammation in the body. Lastly, our study shows that the predictive role of NPR in ECT efficacy varies depending on the state. However, the potential mechanisms underlying this difference need further investigation, including potential neural correlates, which should be studied in future research.

## Conclusions

Overall, our study demonstrated a correlation between baseline NPR levels in adolescent MDD patients and the efficacy of ECT. Compared with other blood index ratios, NPR has a higher predictive value. Especially in female or overweight adolescent patients with high baseline NPR levels, more attention should be paid to determining treatment plans and providing earlier clinical intervention. In conclusion, NPR, as an indicator with relatively effective, convent, minimally invasive, and economical advantages, can be a potential candidate indicator for predicting ECT efficacy and help monitor the treatment of adolescent MDD patients.

## Data Availability

The raw data supporting the conclusions of this article will be made available by the authors, without undue reservation.
